# Eye movement feedback fails to improve visual search performance

**DOI:** 10.1186/s41235-017-0083-2

**Published:** 2017-11-22

**Authors:** Chad Peltier, Mark W. Becker

**Affiliations:** Department of Psychology, Michigan State University Psychology Building, 316 Physics Room 298C, East Lansing, MI 48824 USA

**Keywords:** Target prevalence, Visual search, Visual attention, Feedback

## Abstract

Many real-world searches (e.g., radiology and baggage screening) have rare targets. When targets are rare, observers perform rapid, incomplete searches, leading to higher miss rates. To improve search for rare (10% prevalence) targets, we provided eye movement feedback (EMF) to observers during their searches. Although the nature of the EMF varied across experiments, each method informed observers about the regions of the display that had not yet been inspected. We hypothesized that feedback would help guide attention to unsearched areas and increase the proportion of the display searched before making a target-absent response, thereby increasing accuracy. An eye tracker was used to mark fixated areas by either removing a semiopaque gray overlay (Experiments 1 and 4) as portions of the display were fixated or by adding the overlay once the eye left a segment of the image (Experiments 2 and 4). Experiment 3 provided automated EMF, such that a new region was uncovered every 540 milliseconds. Across experiments, we varied whether people searched for “Waldo” in images from “Where’s Waldo?” search books or searched for a T among offset Ls. We found weak evidence that EMF improves accuracy in Experiment 1. However, in the remaining experiments, EMF had no effect (Experiment 4), or even reduced accuracy (Experiments 2 and 3). We conclude that the one positive result we found is likely a Type I error and that the EMF method that we used is unlikely to improve visual search performance.

**Electronic supplementary material:**

The online version of this article (doi:10.1186/s41235-017-0083-2) contains supplementary material, which is available to authorized users.

## Significance

In real-world search tasks, such as baggage screening, radiology, and surveillance, the absence of a potential threat (e.g., a gun, cancer, or enemy base) is never certain. This leaves observers lacking knowledge regarding their performance because they cannot know when or how frequently they have missed a target. Yet, in laboratory-based studies of visual search, feedback that a target was missed is a critical cue to adapt search strategies (Chun & Wolfe, 1996). Because this cue is unavailable in real-world scenarios, we attempted to develop a new form of feedback, eye movement feedback (EMF), to help adapt search strategies. EMF uses eye tracking to visually mark areas that the observer has already searched for a potential target. By marking previously inspected areas, EMF’s goal is to make the observer aware of the unsearched areas of an image, thus guiding their attention to new areas for which a target may be present. If effective, EMF could reduce selection errors or failures to inspect a target before deciding a target is absent, which are the most common form of misses (Peltier & Becker, 2016a). We evaluated the effectiveness of EMF through four experiments where we compared rare target detection rates between EMF and control groups. Despite using different forms of EMF and search stimuli, we found that EMF rarely increased accuracy and in some cases actually reduced it. We conclude that EMF is unlikely to be an effective tool to improve visual search performance.

## Background

Radiologists miss as many as 30% of cancers in their examinations (Berlin, 1994; Bird, Wallace, & Yankaskas, 1992). Eye-tracking studies indicate that more than one-third of these misses are the result of an incomplete search in which the radiologist fails to fixate the area around the cancer (Bird et al., 1992; Krupinski, 1995). One possible reason why misses are so high is because cancers are quite rare in scans.

Research suggests that miss rates are far higher for low- than for high-prevalence search targets (Ishibashi, Kita, & Wolfe, 2012; Mitroff & Biggs, 2014; Rich et al., 2008; Schwark, Sandry, Macdonald, & Dolgov, 2012; Wolfe et al., 2007; Wolfe, Horowitz, & Kenner, 2005). Similarly to what occurs in radiology, many of these misses are selection errors (Peltier & Becker, 2016a) whereby the observer fails to inspect the target before responding target absent. To explain this pattern, researchers have posited that rare targets result in low quitting thresholds (Wolfe et al., 2005; Wolfe & Van Wert, 2010). With low quitting thresholds, observers inspect less of the display before responding that the target is absent, resulting in faster reaction times but many misses (Gur et al., 2003; Rich et al., 2008; Schwark et al., 2012; Schwark, Macdonald, Sandry, & Dolgov, 2013; Schwark, Sandry, & Dolgov, 2013; Van Wert, Horowitz, & Wolfe, 2009; Wolfe et al., 2005; Wolfe et al., 2007).

Because most errors are caused by an incomplete search (Peltier & Becker, 2016a), a manipulation that delays search termination might entice observers to perform a more thorough search, thereby increasing the hit rate. On the basis of this logic, Wolfe et al. (2007) attempted to alleviate the low prevalence effect (LPE) by giving “speeding tickets” when responses were made too quickly. This manipulation successfully increased latency to absent responses. Unexpectedly, there was no change in accuracy, criterion, or sensitivity, despite the increased response time. One possible explanation for this pattern of results is that observers made an internal target-absent decision and ceased active search but delayed making a target-absent response to avoid a speeding ticket. These results suggest that simple delay manipulations are particularly ineffective; searches take longer but are no more accurate.

Wolfe et al. (2007) conducted seven experiments and found only one technique that was successful in increasing hit rates in low-prevalence searches. This technique involved interspersing low-prevalence search blocks without feedback with blocks of high-prevalence trials with trial-by-trial performance-based feedback (information about correct or incorrect responses). Although this approach increased target detections in the low-prevalence search blocks, sensitivity did not increase. Instead, the increased hit rate was accompanied by increased false alarm rates, a signature of a shift toward a more liberal decision criterion or an informed increase in target-present guessing as prevalence increases (Peltier & Becker, 2017a, 2017b). This type of criterion shift may not be desirable, particularly because there are many opportunities for false alarms in a low-prevalence search. If this method were applied to a real-world situation, observers’ workloads would increase to allow for “dummy blocks” of high-prevalence searches, and the manipulation would result in a higher proportion of false alarms. Ideally, a manipulation that reduces the LPE would increase hits without an increase in false alarms.

Although Wolfe et al.’s (2007) interspersed high-prevalence block experiment did not achieve this ideal, it did demonstrate the important role that feedback can play in setting quitting thresholds. Indeed, according to an influential model of quitting thresholds, feedback about misses is critical to adaptably setting quitting thresholds (Chun & Wolfe, 1996; Danielmeier & Ullsperger, 2011). Under this theory, feedback about a missed target provides information that the previous search was not adequate, thereby increasing quitting thresholds. In low-prevalence and real-world search scenarios, there may be insufficient feedback about misses to set optimal quitting thresholds. In low-prevalence search tasks, the sparse targets result in few opportunities for feedback about misses. In real-world search tasks, the ability to give accurate feedback about one’s performance is often unavailable because the ground truth is unknown.

Given the important role that feedback can play in setting quitting thresholds, as well as the problems associated with providing performance-based feedback (feedback that a target was missed) in low-prevalence and real-world search scenarios, here we attempt to improve search by providing a different form of feedback, namely “eye movement feedback (EMF),” that provides real-time feedback about an observer’s scanning pattern, allowing an observer to see what aspects of the scene they have and have not searched. The ability to provide this type of EMF requires no foreknowledge of a target’s presence or absence and is not impacted by target prevalence rates. Even so, it may act as a substitute for performance-based feedback and may influence quitting thresholds to make searches more complete.

To investigate this possibility, across four experiments, we provided observers with EMF while they searched for rare targets. Three of these experiments used an eye tracker to provide real-time feedback about the portions of the scene that had been inspected during the trial. A fourth automatically revealed sections of the display one at a time, thereby providing observers information about the amount of the scene that had not yet been searched. Although our initial experiment showed promise for the EMF method, across the experiments and manipulations, it became clear that the EMF approach is not a panacea that mitigates the high miss rates that occur when search targets are rare.

## Methods

### Sample sizes

Across the nine conditions in all experiments, we recruited a total of 369 observers (~41 per condition) from the psychology department’s undergraduate observer pool (ages ranged from 18 to 22 years). Observers received course credit or extra credit. To determine required sample sizes, we estimated (Thalheimer & Cook, 2002) the effect size reported in Wolfe et al.’s (2007) Experiment 7—one of the only studies with a manipulation that reduced miss errors in a low-prevalence search task. That estimated effect size was 1.62. Given that our methods and theirs differed substantially and our desire to be conservative, we based our sample size on the desire to be able to find an effect size that was half of theirs (.81) with power of .95, yielding a target total sample size of 41 observers per condition (see Table 1 for number of observers and stimuli used for each experiment). Observers did not complete the experiment if there was an inability to calibrate the eye tracker (*n* = 9 [2.4%]), and data from participants whose hit or false alarm rate was more than 3 SD from the mean for that experiment were eliminated from further analysis (*n* = 14 [3.8%]).

**Table 1:** Experiment Overview

Experiment	No. of Observers		Images Used	Feedback Type
	No Feedback	Feedback		
1	41	35	Where’s Waldo?	Reveal when fixated
2	29	36	Where’s Waldo?	Occlude after viewing
3	41	44	Where’s Waldo?	Autoreveal
4a	35^a^	41	T among Ls	Occlude after viewing
4b	35^a^	39	T among Ls	Reveal when fixated

^a^ *Note*: This condition is repeated in the table for consistency but represents a single condition in Experiment 4

### Data routines

For each condition, we calculated the percentage of hits and false alarms and the corrected hit rate (percent hits minus percent false alarms). Data analysis was performed on corrected hit rates. For reaction time, we calculated the mean and SD for each observer and eliminated trials with a reaction time greater than 3 SD from the observer’s mean, then we calculated a new mean based on the remaining trials that was used for analyses.

We performed planned independent samples *t* tests comparing the feedback groups with the no-feedback control groups for each experiment. In addition, we calculated the Bayes factor (using the Jeffrey-Zellner-Siow Prior and an unbiased scale *r* of 1) for each comparison using an online Bayes factor calculator (Rouder, Speckman, Sun, Morey, & Iverson, 2009).

**Experiment 1** - The experiment was programmed using Experiment Builder software (SR Research, Ottawa, ON, Canada) for the EyeLink 1000 (SR Research) and was run on a computer with a 20-inch flat panel monitor set at 800×600-pixel resolution with a 75-Hz refresh rate viewed from a head rest 55 cm from the screen. The experiment began with the eye tracker’s calibration process. If the calibration was successful, observers were eye tracked for the duration of the experiment. Stimuli consisted of 18 unique “Where’s Waldo?” images. Adobe PhotoShop (Adobe Systems, San Jose, CA, USA) was used to remove Waldo from each image and replace him with other parts of the scene (*see* Additional file 1: Figure S1, Additional file 2: Figure S2, Additional file 3: Figure S3, and Additional file 4: Figure S4 for examples). Image artifacts from this process should have been consistent across the feedback and control conditions. For target-present trials, an image of Waldo (1×2 degrees of visual angle) was inserted in a random location within nine of the scenes (with the provision that Waldo did not straddle the boundary between image segments). The 90 trials were comprised of a randomly ordered presentation of 81 target-absent trials, which were randomly selected with replacements from among the 18 target-absent scenes and the nine target-present trials. Because of observers’ potential prior expectations that Waldo is always present in a “Where’s Waldo?” search, they received both written and verbal instructions that Waldo was not always present, and they were to make a button response to indicate Waldo was either present or absent from the display. Feedback was manipulated between observers, and the stimuli were identical in the feedback and control conditions.

**Fig. 1:**
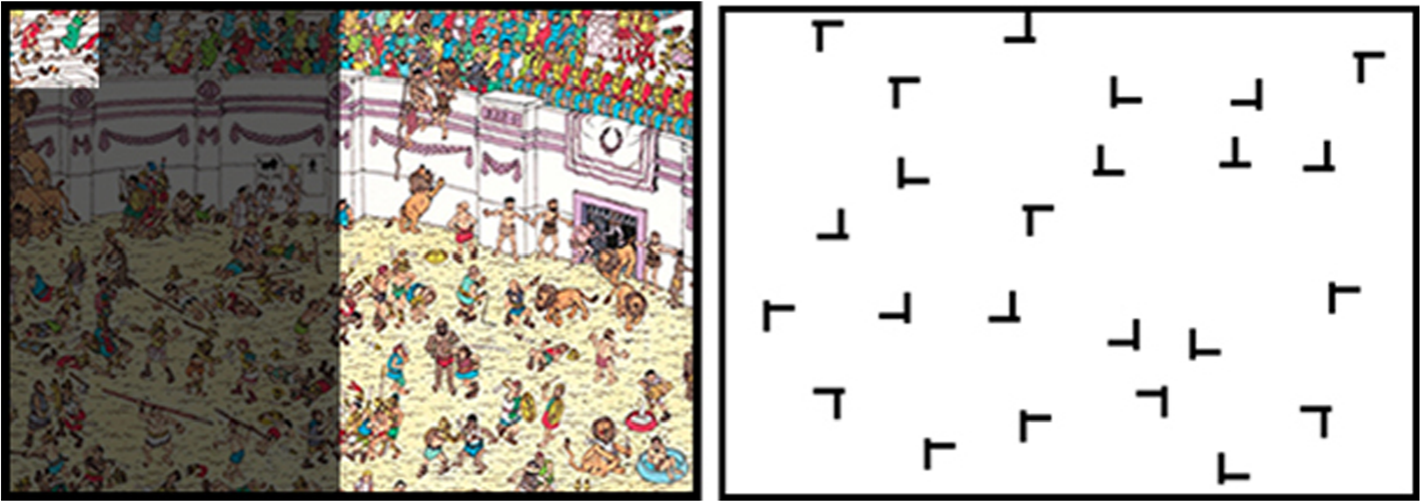
Examples of stimuli used in Experiments 1–3 (*left*) and Experiment 4 (*right*). In the *left panel*, we also provide an example of what the opaque overlay looked like. The *left side* of the figure is what the images looked like with the overlay, and the *right side* of the figure is how images appeared without the overlay. We also showed one segment in the upper left corner without the overlay to give a sense of how large each segment was

Each trial began with a central fixation point that was used to check the calibration of the eye tracker. The task was to search for Waldo and make a target-present/target-absent judgment (via button press) that ended the trial. Each image was segmented into 48 interest areas (each 5.7 degrees × 4.7 degrees) in an 8×6 grid, which was invisible to observers. In the control condition, observers performed a standard search for Waldo. In the experimental condition, observers received EMF. For these observers, the image was first presented with a semiopaque gray overlay, created in Adobe PhotoShop by adding a black (red-green-blue values set to 0) layer with an opacity value of 65%. After initiating a fixation within an interest area, the gray overlay for that segment was removed to reveal the full-color image (*see* Fig. 1). There was no performance-based (correct/incorrect) feedback given to either group.

**Experiment 2 -** The methods used in Experiment 2 were identical to those in Experiment 1, with the following exceptions. The display began with a normal, full-color “Where’s Waldo?” image. Once the eye fixated within a segment and then performed a saccade out of that segment, the segment was covered with the semiopaque gray. In other words, the EMF would be added to a previously inspected interest area only when a new interest area was inspected. In theory, this method allowed for more guidance of search than the method in Experiment 1, because here areas in the periphery (that had not been inspected) appeared without being degraded by the overlay. This method provided feedback about the areas that had already been visited (by graying them out).

**Experiment 3** - The methods used in Experiment 3 were similar to those used in Experiment 1, with the following exceptions. The experiment was programmed using E-Prime 2.0 (Psychology Software Tools, Sharpsburg, PA, USA) and did not use eye tracking. Instead, at the beginning of the trial, the image was presented with the gray overlay. Then each segment of the image was automatically revealed (by removing the gray overlay of that segment) following a reading pattern (from upper left to lower right). Each of the 48 interest areas’ semiopaque overlays were removed one at a time every 540 milliseconds. This timing was chosen using 1/48th (for the 48 interest areas) of the average reaction time in correct target-present trials from the feedback group in Experiment 1. Trials were self-terminated with a target-present or target-absent response. This method was chosen to determine whether the benefits of removing a gray overlay could be obtained in a manner that did not require the expense of eye tracking. The removal of the gray overlay still allowed observers to determine how much of the scene had not yet been fully inspected (i.e., revealed).

**Experiment 4** –For Experiment 4, we changed the search task from a “Where’s Waldo?” search to a search for a T among offset Ls (*see* Fig. 1). Our rationale for doing so was that the “Where’s Waldo?” images were extremely cluttered, and thus multiple objects appeared in each of our predefined image segments. This raised the possibility that observers may have inspected some but not all of the objects within a segment before moving to a new segment of the image. If EMF encouraged observers to move to uninspected regions of the scene and discouraged returning to previously inspected areas, the feedback may have hindered target detection when Waldo was not inspected during the initial investigation of a scene segment. These new T-among-L stimuli avoided this concern by presenting a single stimulus in each segment of the scene.

In Experiment 4, the task was to search among 24 items (1.2 degrees × 1.2 degrees of visual angle) for a T in an array of offset Ls and respond present or absent via button press (*see* Fig. 1). In 18 of 180 trials (10% target prevalence), one randomly chosen L was replaced with a T. Each item was randomly rotated to 0, 90, 180, or 270 degrees from vertical. Each search array was divided into 24 (a 6×4 matrix) equal-sized (6.4 degrees × 7.1 degrees) interest areas. A single item was placed within each region with jitter. These interest areas also served as the basis for EMF.

One group received a control condition within normal search stimuli. A second condition (reveal) was analogous to that in Experiment 1: The display began with the semiopaque gray overlay, and the gray for a segment was removed upon fixation within that segment. The final condition (masking) was analogous to that of Experiment 2: The display began without a gray overlay, and after the eye fixated within a segment and then moved to a new segment, the previously inspected region was overlaid with the semiopaque gray.

## Results and discussion

**Fig. 2:**
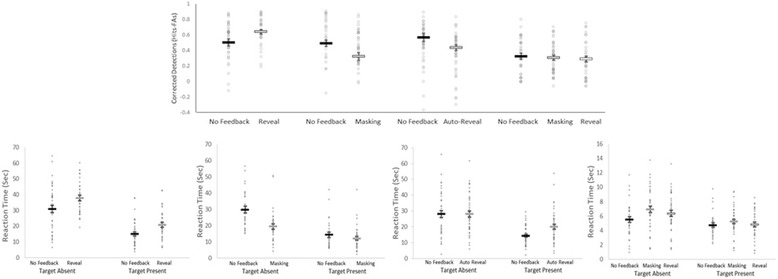
The *top panel* presents the corrected accuracy (percent hits minus percent false alarms) for each experiment and condition. The *small gray circles* are individual subjects’ data, the *wide markers* are the mean values, and *error bars* represent the SEM. Means for the control conditions are presented in *black*, and the means for the various feedback conditions are presented in *gray*. The *bottom panel* presents the mean reaction times for the correct target-absent responses for each experiment and condition. The *small gray circles* are individual subjects’ data, the *wide markers* are the mean values, and *error bars* represent the SEM. Mean values for the control conditions are presented in *black*, and the mean values for the various feedback conditions are presented in *gray. FAs* False alarms

Table 2 (accuracy) and Table 3 (reaction time) present the summary statistics and statistical comparisons for each experiment. The data are also graphically presented in Fig. 2.

**Table 2:** Accuracy as a Function of Experiment and Condition

Experiment	Hit Rate		FA Rate		Corrected Accuracy (Hits − FAs)		Independent Samples *t* Test (Two-tailed)	Bayes Factor	Strength of Evidence^a^	Effect of Feedback
	No Feedback	Feedback	No Feedback	Feedback	No Feedback	Feedback				
1	65.3% (3.8%)	75.5% (2.8%)	15.2% (2.6%)	11.6% (1.5%)	50.1% (4.6%)	63.9% (3.1%)	*t*(74) = −2.43, *p* = .02	Support for H_1_ 2.48	Weak	Improved performance
2	65.9% (4.4%)	53.4% (3.8%)	9.2% (1.4%)	10.0% (2.4%)	56.7% (4.9%)	43.4% (3.8%)	*t*(63) = 2.19, *p* = .03	Support for H_1_ 1.60	Weak	Hindered performance
3	71.0% (3.6%)	59.1% (2.7%)	21.9% (2.2%)	27.0% (3.5%)	49.1% (4.1%)	32.1% (5.0%)	*t*(83) = 2.60, *p* = .01	Support for H_1_ 3.52	Some	Hindered performance
4a	34.7% (4.1%)	32.2% (3.6%)	2.3% (1.8%)	3.2% (1.7%)	32.4% (3.9%)^b^	29.0% (3.7%)	*t*(72) = .646, *p* = .52	Support for H_0_ 5.85	Some	No effect
4b	34.7% (4.1%)	36.9% (2.8%)	2.3% (1.8%)	6.4% (2.3%)	32.4% (3.9%)^b^	30.5% (3.2%)	*t*(74) = .393, *p* = .70	Support for H_0_ 4.66	Some	No effect

*FA* False alarm

^a^Criterion based on Jeffreys (1961) as cited in Rouder et al. (2009)

^b^ *Note*: This condition is repeated in the table for consistency but represents a single condition in Experiment 4

**Table 3:** Reaction Times for Correct Target-Absent Trials as a Function of Experiment and Condition

Experiment	Target-Present RT (Seconds)		Target-Absent RT (Seconds)		Independent Samples *t* Test (Two-tailed)	Bayes Factor	Strength of Evidence^a^	Effect of Feedback
	No Feedback	Feedback	No Feedback	Feedback				
1	15.11 (1.30)	20.84 (1.58)	30.79 (2.26)	37.84 (1.77)	*t*(74) = −2.40, *p* = .02	Support for H_1_ 2.31	Weak	Increased quitting threshold
2	14.26 (1.67)	12.70 (1.24)	29.76 (2.15)	19.41 (1.64)	*t*(63) = 3.90, *p* < .001	Support for H_1_ 107.16	Strong	Decreased quitting threshold
3	14.28 (0.97)	20.06 (1.55)	28.07 (2.16)	27.99 (1.87)	*t*(83) = 0.03, *p* = .98	Support for H_0_ 6.02	Some	Had no effect
4a	4.87 (3.36)	5.62 (3.69)	5.52 (0.41)^b^	6.37 (0.44)	*t*(72) = −1.42, *p* = .16	Support for H_0_ 2.25	Weak	Had no effect
4b	4.87 (3.36)	6.01 (3.36)	5.52 (0.41)^b^	6.92 (0.45)	*t*(76) = −2.27, *p* = .03	Support for H_1_ 1.77	Weak	Increased quitting threshold

*RT* Reaction time

^a^Criterion based on Jeffreys (1961) as cited in Rouder et al. (2009)

^b^ *Note*: This condition is repeated in the table for consistency but represents a single condition in Experiment 4

Experiment 1 suggested that EMF might be an effective method of reducing errors in search tasks with rare (10% prevalence) targets. The feedback condition showed better detection rates than the control condition. In addition, prior to responding target absent, observers performed longer and more complete searches in the feedback than the control condition. The feedback group had significantly longer reaction times before responding target absent (Table 3) than the control group, showing that receiving EMF delays quitting time. The feedback group also visited significantly more interest areas [*t*(73) = 3.41, *p* < 0.001, Cohen’s *d =* .80, a large effect size] than the control group before responding target absent.

In short, the data from Experiment 1 were encouraging. We note, however, that on the basis of the Bayes factor, the alternative hypothesis for both accuracy and reaction time were only ~ 2.4 times more likely than the null hypothesis, an OR that is classified as weak evidence by convention (Rouder et al., 2009). Even so, we were encouraged and performed additional experiments to determine whether we could replicate and extend the results.

In Experiment 2 (with the masking feedback), the pattern of results completely reversed. That is, the feedback group showed worse target detection performance and faster target-absent responses than the control group. These data suggest that feedback led to decreasing quitting thresholds and target detection. Similarly, in Experiment 3, the autoreveal method of providing EMF also hindered target detection relative to the control. In terms of reaction time, there was no evidence that this autoreveal technique altered quitting thresholds. In short, Experiment 2 and 3 suggested that the findings from Experiment 1 may have been a Type I error.

Before accepting that conclusion, however, we reasoned that the methods of providing feedback in Experiments 2 and 3 may have impaired target detection because they did not encourage people to adequately inspect each region. Perhaps covering a segment once the eye had moved away from it (Experiment 2) discouraged refixations on that segment. Thus, if people inspected one object in the segment (but not Waldo) and then moved to a new segment, they would be unlikely to revisit the location and would thus fail to return to find Waldo. Consistent with this interpretation, we did find fewer refixations [*t*(63) = 11.03, *p* < .001] on a segment in the feedback (M = 13%, SEM = 0.1%) than in the control condition (M = 30%, SEM = 1.2%). Similarly, the autoreveal method may have encouraged people to keep “moving forward” as new segments were revealed, thereby reducing refixations. Again, if Waldo was not one of the first items inspected within a region, the observer may have moved on to new segments prior to identifying Waldo and may have been discouraged by the method from returning to previously viewed segments for additional analysis. Although we did not have eye movement data to validate this lack of refixation account for Experiment 3, this explanation seemed plausible.

Thus, we ran Experiment 4, in which we simplified the displays (by switching to a T-among-Ls search) and had a single item appear in each segment. This change in stimuli should eliminate the problems associated with fixating within a segment but not inspecting the actual target, because only one item appeared in each segment. In Experiment 4, neither the revealing nor the masking method of providing EMF improved target detection relative to control. In addition, the reaction time data suggest that revealing may have delayed reaction times, consistent with a raising of the quitting threshold, but there was no evidence for a raising of quitting thresholds with the masking method.

## Conclusions

What conclusions can we draw from this mixed bag of results? First, we can say with some confidence that EMF is not a panacea that eliminates the high miss rates associated with a low-prevalence search. Although we found one case (Experiment 1) where it appeared to help, that result seems to be the exception rather than the rule. Making slight modifications to the type of feedback (Experiments 2 and 3) or the type of stimuli (Experiment 4) showed no benefit of EMF and in some cases showed a cost of doing so. In short, our results suggest that providing EMF is not likely to improve rare target detection.

It is worth noting that, at the outset, we truly thought that this was a promising approach, and we wanted to find evidence that EMF could help. Drew and Williams (in press) also believed this method might be effective. However, across a number of attempts, they also found little evidence that EMF improved performance (*also see* Dickinson & Zelinsky, 2005).

Across the present experiments, as well as those of Drew and Williams, a wide variety of feedback approaches have been tried; stimuli and search difficulty have varied widely; and the consensus of the results as a whole is that this type of EMF is an ineffective way of reducing the high miss rates in low-prevalence searches.

As such, the work we present here, as well as that of Drew and Williams, adds to a growing body of work that highlights how robust and stubborn the low-prevalence effect is (Kunar, Rich, & Wolfe, 2010; Schwark, Macdonald, et al., 2013; Wolfe et al., 2007). Although there were solid theoretical bases to believe that EMF may improve search for rare search targets (Hout, Walenchok, Goldinger, & Wolfe, 2015; Peltier & Becker, 2016a), visual search performance seems unlikely to be improved by these manipulations. At the theoretical level, this suggests that the quitting thresholds and decision criteria that have been deemed responsible for the low-prevalence effect (Hout et al., 2015; Peltier & Becker, 2016a; Wolfe & Van Wert, 2010), although sensitive to target prevalence, may be insensitive to other types of manipulations.

At a practical level, these results offer two conclusions. First, we would not recommend that researchers attempt to implement this type of feedback in hopes of improving rare target detections. On the whole, the evidence suggests that doing so is unlikely to improve performance, and depending on how the manipulation is performed, it may even hinder performance. Second, we would advise that researchers who are interested in discovering ways of improving visual search performance for rare targets explore other techniques. Across both our sets of experiments and those of Drew and Williams, we believe the space of possible EMF interventions has been well probed. Indeed, in our laboratory, we have moved on, and rather than finding ways to modify the search tasks to improve low-prevalence search performance, we have had success (Peltier & Becker, 2016b, 2017a) investigating individual differences and developing a screener that could be used to identify those individuals who are likely to have high target detection rates, even in the face of low target prevalence. That approach has been more successful in both our laboratory (Peltier & Becker, 2016b, 2017a, 2017b) and another (Schwark, Sandry, et al., 2013).

Finally, it is worth discussing the fact that some of our manipulations appear to reduce target detection rates. Our belief is that some of these manipulations (those in Experiments 2 and 3) may have encouraged people to move rapidly from section to section without revisiting previously inspected segments. Given that low prevalence shifts the decision criterion for evaluating an object as a target higher (Peltier & Becker, 2016a; Wolfe & Van Wert, 2010), the reduced time spent inspecting a segment may lead to more identification errors (inspecting an object but not identifying it as a target). Consistent with this speculation, we have previously reported that successful target detection requires longer fixation on the target when targets are rare (Peltier & Becker, 2016a, 2016b). If an EMF method reduces the time spent viewing each item, it may increase these types of identification errors.

Despite our best efforts to develop a method of providing real-time feedback about the search process (i.e., which areas have and have not been inspected) to improve rare target search, we conclude that these EMF manipulations, as we have implemented them here, are unlikely to improve rare target search performance and may even hinder it. Taken together with the independent results of Drew and Williams, we believe we have relatively compelling evidence that attempting to improve rare target search with EMF is an unfruitful approach.

## Additional files

Additional file 1: Figure S1. "Where's Waldo?" example image one of two with the target Waldo removed and replaced by a non-target. (JPG 686 kb)

Additional file 2: Figure S2. "Where's Waldo?" example image one of two with the target Waldo in place. (JPG 677 kb)

Additional file 3: Figure S2. "Where's Waldo?" example image two of two with the target Waldo removed and replaced by a non-target. (JPG 810 kb)

Additional file 4: Figure S4. "Where's Waldo?" example image two of two with the target Waldo in place. (JPG 810 kb)
