# Supplementary figures and images for: Eye movement feedback fails to improve visual search performance
Source: Cogn Res Princ Implic. 2017 Nov 22;2:47. doi: 10.1186/s41235-017-0083-2 (PMC5698387; doi:10.1186/s41235-017-0083-2)

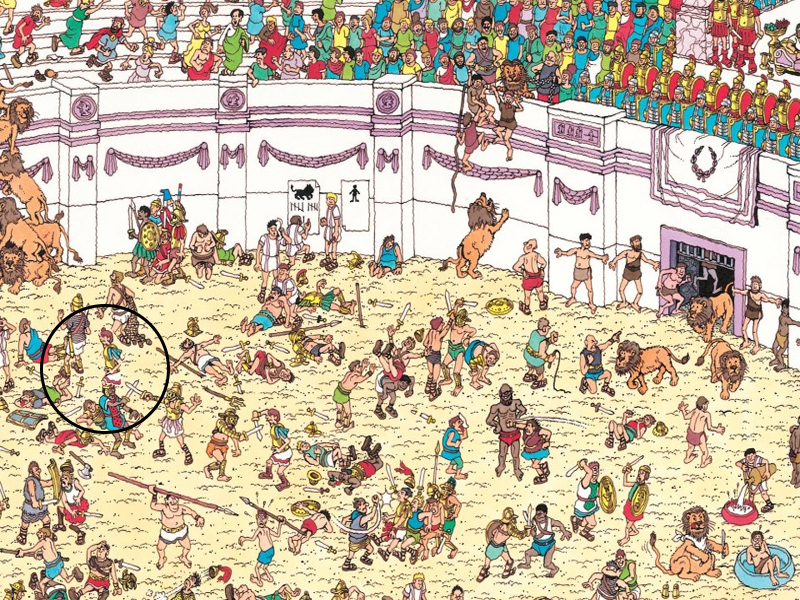

Supplement: Supplementary file 1 — "Where's Waldo?" example image one of two with the target Waldo removed and replaced by a non-target. (JPG 686 kb) [file 41235_2017_83_MOESM1_ESM.jpg]

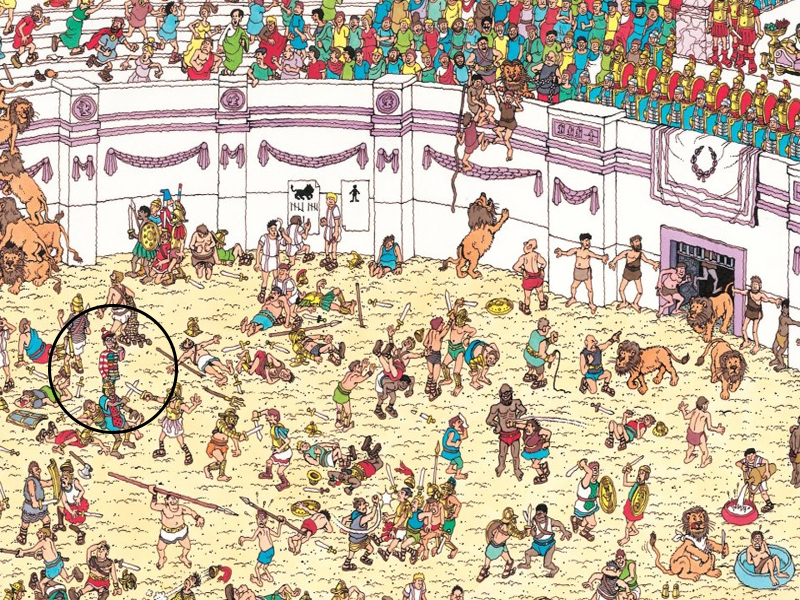

Supplement: Supplementary file 2 — "Where's Waldo?" example image one of two with the target Waldo in place. (JPG 677 kb) [file 41235_2017_83_MOESM2_ESM.jpg]

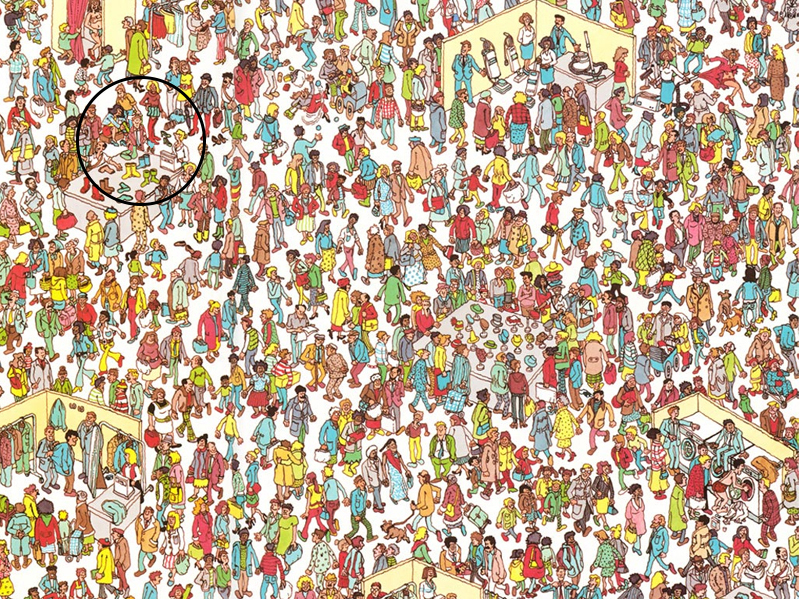

Supplement: Supplementary file 3 — "Where's Waldo?" example image two of two with the target Waldo removed and replaced by a non-target. (JPG 810 kb) [file 41235_2017_83_MOESM3_ESM.jpg]

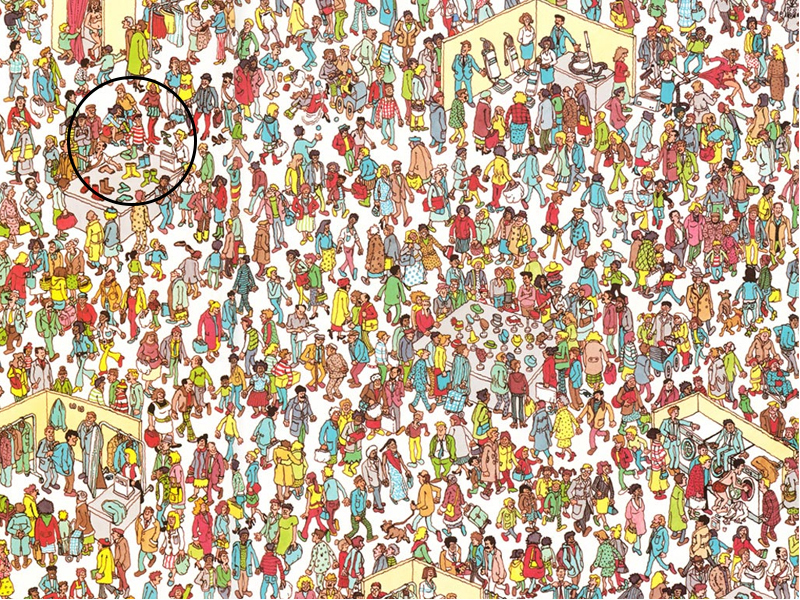

Supplement: Supplementary file 4 — "Where's Waldo?" example image two of two with the target Waldo in place. (JPG 810 kb) [file 41235_2017_83_MOESM4_ESM.jpg]
